# Fixator-Assisted Nailing for Femur Neck Fracture Nonunion: A Case Series Study

**DOI:** 10.1155/2022/5676144

**Published:** 2022-04-14

**Authors:** Majdi Hashem, Mohammad S. Al-Tuwaijri

**Affiliations:** ^1^Department of Surgery, Faculty of Medicine, Imam Mohammad Ibn Saud Islamic University, Riyadh, Saudi Arabia; ^2^Orthopedic Department, Prince Mohammad Bin Abdulaziz Hospital, Riyadh, Saudi Arabia

## Abstract

**Background:**

Femoral neck fractures in young adults tend to be a result of high-energy trauma with a common pattern of Pauwels type III fracture, and they require timely and meticulous diagnosis and management. The objective of this study was to assess the clinical and radiological outcomes of the fixator-assisted nailing technique for managing femur neck fracture nonunion*. Methods*. This was a case series study of 16 patients with nonunion femoral neck fractures treated via a fixator-assisted nailing technique. Our inclusion criteria comprised the inclusion of any patient between the ages of 14 and 60 years old with a neglected neck of femur fracture or nonunion of the femur neck. In addition, we only included patients without further posttreatment trauma and without known metabolic diseases. The conditions that were excluded from this study included hip joints with preexisting osteoarthritis, radiographic evidence of avascular necrosis of the femoral head, and associated ipsilateral acetabulum fracture or fracture-dislocation. The fracture characteristics that were selected for the fixator-assisted nailing (FAN) technique were clear signs of pseudoarthrosis (such as sclerosis, clear fracture line defects, and failure of implants), in addition to evidence of varus malalignment. All fractures were Pauwels type III. Radiographs of the pelvis with both hips and a posteroanterior (PA) view of the injured hip were taken. Full weight bearing was allowed in all the patients from the first day postoperatively. Physical therapy was started for pain reduction modalities, stretching, and abductor strengthening.

**Results:**

Union of the femur neck fracture and osteotomy site was achieved in all patients. An excellent functional status after four months of follow-up was found based on a modified Harris hip score questionnaire. At follow-up, no patient was suffering from pain or flexion contracture. Preoperative limb length discrepancy (LLD) (cm) was 1.8 ± 0.8 cm and postoperative was 0 ± 0.1 cm, *p* < 0.001. Preoperative neck-shaft angle (NSA) (*o*) was 85.6 ± 4.4 and postoperative was 126.9 ± 2.5, *p* < 0.001. Preoperative Pauwels angle (*o*) was an average of 50.4 ± 5.9 and postoperative was 31.3 ± 2.5, *p* < 0.001.

**Conclusion:**

Our study indicates that FAN has a high success rate in young patients with nonunited femoral neck fractures.

## 1. Introduction

Femoral neck fractures in young adults tend, unlike in older people, to be a result of high-energy trauma with a common pattern of Pauwels type III fracture, and they require timely and meticulous diagnosis and management [1–4]. Anatomical reduction and stable internal fixation are essential for achieving the goals of treatment in this young population, allowing preservation of the femoral head while minimising the rates of nonunion and osteonecrosis [1]. A delayed presentation of fracture of the femoral neck is one where there is a delay of 48 hours to 20 days between injury and diagnosis; whereas, in a neglected fracture, this delay is more than 21 days [2]. One of the most difficult fractures to treat in young people is the neglected fracture, which has the most at stake [3].

Pauwels' classification, which was introduced in 1935, was the first biomechanical classification for femoral neck fractures [5]. The calculated angle determines the balance between shearing stress and compressive force at the fracture line. In type III, the angle is more than 50; the shearing force is predominant and is associated with a significant amount of varus force, which will more likely result in fracture displacement and varus collapse [5, 6]. Fractures with high vertical angles (Pauwels III) are more prone to nonunion owing to high shearing forces [7].

The two major complications of femoral neck fractures are avascular necrosis (AVN) of the femoral head and nonunion. Nonunion has a reported incidence of 10–34% [2, 3]. Subtrochanteric valgus osteotomy fixed with an angle blade plate or a dynamic condylar screw has been the treatment of choice over recent years [1, 3, 4, 7, 8]. In addition to its technical difficulty, invasiveness, and prolonged postoperative rehabilitation, it has carried a high nonunion rate up to 20% [8]. This study aims to describe a novel technique for treating femoral neck fracture nonunion.

## 2. Patients and Methods

### 2.1. Study Design

This case series study was conducted at Prince Mohammad bin Abdulaziz Hospital in Riyadh, Saudi Arabia, between December 2015 and December 2019.

### 2.2. Study Procedure

The patients described herein were managed with a novel surgical technique comprising fixator-assisted nailing (FAN) and proximal femoral osteotomy. The patients were informed that the data concerning their case would be submitted for publication, and they provided their consent.

### 2.3. Inclusion/Exclusion Criteria

Our inclusion criteria comprised the inclusion of any patient between the ages of 14 and 60 years old with a neglected neck of femur fracture or nonunion of the femur neck. In addition, we included patients without further posttreatment trauma and the absence of known metabolic diseases. The patients who were excluded from this study included hip joints with preexisting osteoarthritis, radiographic evidence of AVN of the femoral head, and associated ipsilateral acetabulum fracture or fracture-dislocation. The fracture characteristics that were selected for the FAN technique were those with clear signs of pseudoarthrosis (such as sclerosis, clear fracture line defects, and failure of implants) in addition to evidence of varus malalignment.

### 2.4. Clinical Assessment

A clinical assessment was done for the lower extremities to evaluate limb length discrepancy (LLD) using a measuring tape from the anterior superior iliac spine to the medial malleoli. A distal neurovascular assessment was made, and their functional status was measured. Anteroposterior radiographs of the pelvis showing both hips and the anteroposterior/lateral view of the affected hip were taken, requiring measurement of the neck-shaft angle (NSA), and Pauwels angle was obtained. All of the fractures were Pauwels type III. These parameters were reassessed postoperatively (Tables 1 and 2). All the patients consented to surgery, and all were informed about the surgical plan to use FAN with subtrochanteric osteotomy as a developing technique for treating nonunion of a femur neck fracture.

### 2.5. Surgical Technique

The FAN technique was performed at an average of 5–13 months from the index surgery (fracture fixation). The patient was placed in a supine position on a traction table and checked with fluoroscopy from the hip to the knee in both planes before sterile preparation; a fluoroscopic imaging unit was brought in from the contralateral side of the table. The trochanter-head line was high because of varus deformity. To make the nail insertion easier, a pair of external fixator 6 mm Schanz pins was inserted perpendicular to the anatomical axis towards the centre of the femoral head in the frontal plane and posterior in the lateral plane. Another pair of Schanz pins was placed distally perpendicular to the anatomic axis of the distal femur and far from the planned distal tip of the nail or anterior to the nail track to avoid anterior notching in the case of a desired longer nail size or a long femur. Orthofix external fixator (LRS) bars were attached to the proximal and distal Schanz pins, utilising a swivel clamp and a conventional clamp, respectively.

The procedures were executed by two orthopaedic consultants who had experience in deformity and traumatology; the average operative time was 1 hour 50 minutes. All were done under general anaesthesia. There were no intraoperative complications.

### 2.6. Pauwels' Osteotomy

**Figure 1:**
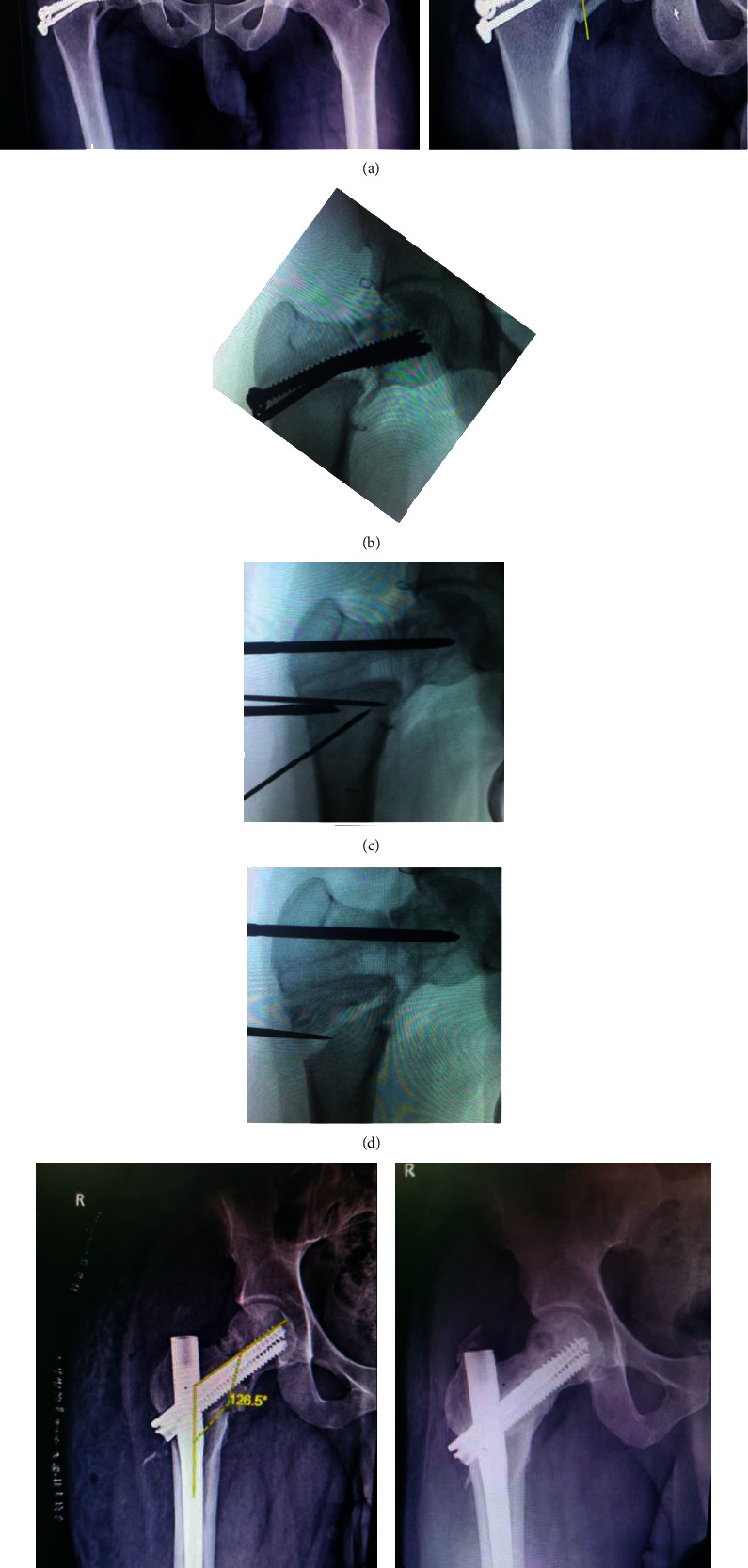
An AP radiograph of the pelvis and right hip reveals a nonunion neck of femur fracture with varus deformity, assessed at 81 degrees Pauwels angle. Hardware that has been bent. (b) Intraoperative image shows pseudoarthrosis with lysis and scelorosis, as well as impending hardware failure. (c) Visualization of Pauwels' osteotomy execution during surgery. (d) The wedge bone was leveraged into the bone defect by 7 mm cannulated screws that were removed. (e)-(f) Radiographs depicting the postoperative outcome and a 12-month follow-up.

**Figure 2:**
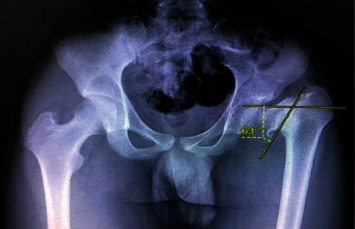
AP radiograph of the pelvis showing pseudoarthrosis of femur neck fracture with significant varus deformity: neck-shaft angle, 75 degree; Pauwels type 3, 63 degree.

**Figure 3:**
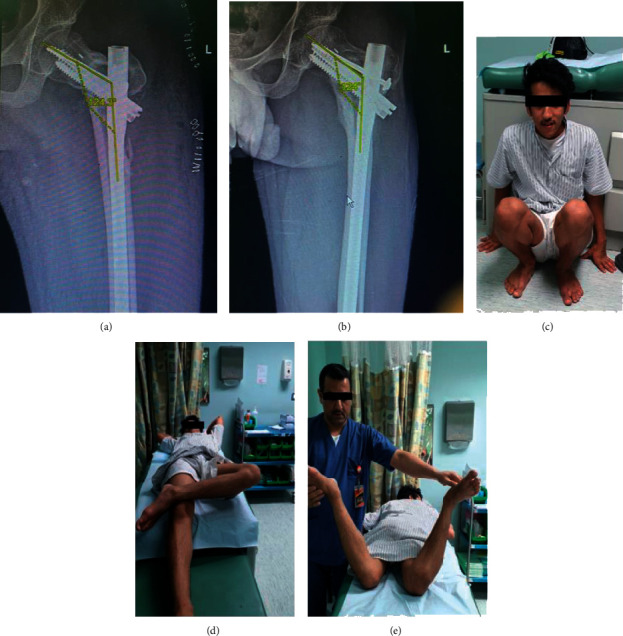
(a) Postoperative radiograph sequence showing correction achieved and the position of implant within the femoral head and intramedullary canal. (b) 6 months postoperative follow-up showing 124 degree neck-shaft angle. (c) 4 months postoperative clinical photo showing the ability of the patient to squat. (d) External rotation (cross leg). (e) Internal rotation.

Pauwels' osteotomy (closing wedge intertrochanteric osteotomy) performed for three hips was executed through the preexisting incision used to remove the screw fixation from the index surgery. The upper base of the triangle was parallel to the proximal pin, while the distal base of the triangle was located in an oblique fashion after measuring a 40 mm lateral triangular base to provide a 40° correction. The oblique distal cut, with the help of the osteotome, allowed us to lever and impact the bone wedge proximally into the squishy cancellous bone around the 7 mm screws, which were removed. This was easily achieved because of the inactivity atrophy after prolonged immobilisation and pain (Figure 1). Meanwhile, Southwick osteotomy was carried out in 13 patients as a transverse cut just below the level of the lesser trochanter (Figures 2 and 3).

Good control of the deformity was achieved by placing this pin centrally into the femoral head connected to a swivel clamp. We found that the swivel clamp allows us only 25° of freedom, which was not enough in most patients. We tried temporarily removing the swivel clamp, blocking the screw, and levering the pin further distally. Optimal correction was achieved, and the swivel clamp was locked onto the bar at the desired position from the outer surface. After obtaining the proper correction, intramedullary nailing was inserted utilising the trochanter entry point to maintain the correction achieved. A small wedge was taken off the lateral cortex of the proximal segment to facilitate nail insertion without disturbing the translation achieved using a small diameter nail (10 mm). The external fixator bar was removed after the distal interlocking screws were in place in all patients.

There was no residual pain. The gait pattern changed from the Trendelenburg pattern, secondary to weak and short hip abductors, to a normal gait pattern.

### 2.7. Postoperative Protocol

Full weight bearing was allowed for all patients from postoperative day one. Physical therapy started for pain reduction modalities, stretching, and abductor strengthening. Follow-up was made at 2, 6, 12, 16, 24, and 48 weeks.

The rehabilitation programme took from 12 to 15 weeks. Most of the patients began walking with the help of a physiotherapist on the first or second day after surgery. The protocol was tailored to restore gait balance, proprioception, strength, and flexibility of the muscles. The therapist focused on the hip joint range of motions, strengthening all surrounding muscles. Follow-up was made at 2, 6, 12, 16, 24, and 48 weeks for clinical and radiological assessment.

### 2.8. Ethical Approval

The study was approved by the Research Ethics Committee at Al-Imam Mohammad Ibn Saud Islamic University, Riyadh, Saudi Arabia (HAFO-01-R-011/15-10-2017), and it followed the National Committee of Bioethics guidelines.

### 2.9. Statistical Analysis

The statistical analyses were carried out using SPSS (version 27). Descriptive statistics were used to describe demographic characteristics. Continuous data were reported as mean ± SD. A paired sample *t*-test was used to explore the difference in the mean LLD, NSA, and Pauwels angle values between patients' pre and postoperation. A two-sided *p* < 0.05 was considered to be statistically significant.

## 3. Results

A total of 16 patients with femoral neck fracture nonunion were treated using the FAN technique. All were secondary to motor vehicle accidents. Their ages ranged from 17 to 44 years, and there were 14 males and 2 females. Union of the femur neck fracture and osteotomy site was achieved in all patients.

### 3.1. Patient Assessment

The clinical union was confirmed when there was a painless hip range of movement and painless full weight bearing. A functional assessment was performed using a modified Harris hip score. Subjective and objective measures were taken at the four-month postoperative follow-up and were excellent (more than 90°) in all patients. Postoperative average hip range of motion: abduction was 30°, flexion was 130°, internal rotation in flexion ranged between 20 and 30° for all patients, and external rotation in flexion ranged between 40 and 45° (Figure 3(c)–3(e)) (Table 1).

### 3.2. Angle of Correction

**Figure 4:**
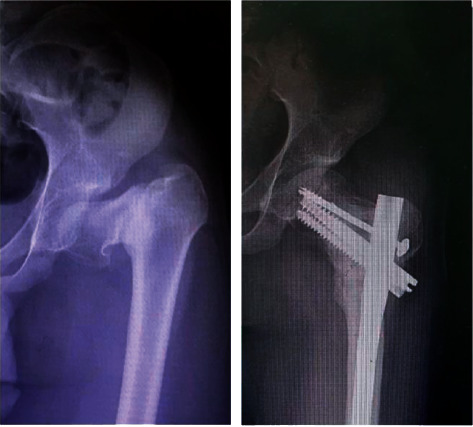
AP radiograph of left hip illustrating the preoperative and postoperative results.

Preoperative LLD (cm) was 1.8 ± 0.8 cm and postoperative was 0 ± 0.1 cm, *p* < 0.001, as given in Table 2. Preoperative NSA (*o*) was 85.6 ± 4.4 and postoperative was 126.9 ± 2.5, *p* < 0.001 (Figure 4). Preoperative Pauwels angle (*o*) ranged from 40 to 63, with an average of 50.4 ± 5.9 and postoperative was 31.3 ± 2.5, *p* < 0.001.

## 4. Discussion

Currently, the treatment options available to manage nonunion of femur neck fractures include refixation, solitary bone grafting, valgus osteotomy, and prosthetic arthroplasty. The valgus intertrochanteric osteotomy (VITO) described by Pauwels carried a high union rate of 80–90%, as described by most authors [5–7, 9], and good to excellent outcomes when it comes to the salvage of failed internal fixation of femoral neck fractures [8, 10, 11]. However, VITO does not come without pitfalls, limitations, and complications. The challenges in this approach are determined by the lack of bone stock within the femur neck and head, the technical demand encountered, especially for Pauwels III, where the required angle of correction reaches up to 50 degrees, the need to extend the osteotomy to the subtrochanteric region, the changes in proximal femoral conformation, and possible abduction and external rotation deformity [12]. VITO, with a valgus angle of over 30 degrees, may also increase the risk of AVN and make subsequent total hip arthroplasty in the future difficult [12]. The reported incidence of AVN post-VITO was 10–40%, which represents a relatively high percentage [8, 13, 14], and nonunion is still reported in 10–20% of the patients [9].

The technical difficulty with different internal fixation types, whether dynamic hip screws or angled blade plates, and their inferior biomechanical stability in comparison to cephalomedullary nails and the previously mentioned shortcoming of VITO suggest recruiting the FAN technique to tackle this challenging condition that we encounter in our practice.

The FAN technique described by Paley et al. in 1997 [15] has become the gold standard for correcting long bone deformities [16, 17]. This technique combines the accuracy, minimal invasiveness, and safety of external fixation with the convenience of internal fixation. The intramedullary nail prevents the recurrence of the deformity, which is especially important in patients with metabolic bone diseases who are prone to recurrence of the deformity as the metabolic problem continues [18]. The FAN technique has gained popularity in the treatment of lower limb deformities, mainly around the knee joint (distal femur and proximal tibia) and distal tibia deformities [19–21]. Several authors have reported the effectiveness of the FAN technique [15–20, 22–24]. Recently, Hashem et al. [25] reported satisfactory results in slipped capital femoral epiphysis sequelae using the FAN technique with a subtrochanteric osteotomy to correct proximal femur deformity (coxa vara, femoral retroversion, symptomatic impingement, and LLD). They published the first reported case of the FAN technique to manage a proximal femoral deformity.

We assessed the FAN technique for femur neck fracture nonunion. The clinical union was confirmed when there was a painless hip range of movement and painless full weight bearing. A functional assessment was performed using subjective and objective data based on a modified Harris hip score. The patients involved in this case series regained excellent functional status. In the follow-up, a loss of correction and recurrence of the deformity were not encountered because of the superior biomechanical stability provided by the retained intramedullary nail.

In the present study, FAN was performed on the nonunited fractured neck of the femur with Pauwels type III varus deformity. Union of the femur neck fracture and osteotomy site was achieved in all patients (100%). Preoperative Pauwels angle was 50.4 ± 5.9 and postoperative was 31.3 ± 2.5. Preoperative NSA was 85.6 ± 4.4 and postoperative was 126.9 ± 2.5. Preoperative LLD was 1.8 ± 0.8 cm and postoperative was 0 ± 0.1 cm. Follow-up revealed no patients with AVN. Among the united fractures, all patients were able to sit cross-legged, squat, and stand on the affected leg, functions that are needed in day-to-day life activities.

This technique reduces the need for large, open approaches, meaning less dissection and soft-tissue damage, bleeding, scarring, and postoperative pain, and it enables rapid rehabilitation compared with an open technique. The FAN approach effectively manages proximal femoral deformities in selected patients. It requires careful analysis of the deformity, meticulous preoperative planning, and surgeon experience with both intramedullary nailing and external fixation techniques. It requires a reasonable learning curve to avoid intraoperative hardware impingement between the fixator and the proximal nail targeting device.

This case series study is among the first to assess the clinical and radiological outcomes of the FAN technique for managing femur neck fracture nonunion. Additionally, it describes a novel approach that tackles technical difficulty with different internal fixation types, whether dynamic hip screws or angled blade plates, and their inferior biomechanical stability in comparison to cephalomedullary nails. A case series study design can help in generating hypotheses that are useful in designing further studies. At the same time, this case series study has limitations. A case series study design cannot establish causal inferences. Additionally, it is susceptible to different types of bias, including selection bias and measurement bias. In addition, our study sample was relatively small. Therefore, our findings should be interpreted carefully.

## 5. Conclusion

Fixator-assisted nailing has a high success rate in young patients with nonunited femoral neck fractures. It is safe and effective, with excellent clinical and radiological outcomes.

## Figures and Tables

**Table 1:** Patients' clinical data.

Postoperative hip range of motion									
Patient number	Age (years)	Index surgery (months)	follow-up (months)	Abduction (*o*)	Flexion (*o*)	Internal rotation in flexion (*o*)	External rotation in flexion (*o*)	Union (months)	Union (osteotomy) (number of months)
1	28	6	24	30	130	20	40	6	4
2	28	4	24	30	130	30	45	6	4
3	17	5	8	30	130	30	45	4	3
4	37	8	24	30	130	20	40	8	5
5	26	12	24	30	130	30	45	6	4
6	26	12	24	30	130	30	45	6	4
7	24	9	24	30	130	20	40	6	4
8	41	5	24	30	130	20	40	7	5
9	20	6	6	30	130	20	35	5	4
10	19	8	24	30	130	30	45	6	4
11	30	9	10	30	130	20	40	6	4
12	20	8	12	30	130	20	40	6	4
13	24	7	12	30	130	20	40	6	4
14	24	20	12	30	130	2 0	35	6	4
15	29	5	6	30	130	30	45	6	5
16	44	6	6	30	130	20	40	6	5

**Table 2:** Patients' clinical data pre and postoperation.

	LLD (cm)		*P* value	NSA (°)		*P* value	Pauwels angle (°)		*P* value
	Pre	Post	**<0.001**	Pre	Post	**<0.001**	Pre	Post	**<0.001**
1	2	0		80	130		57	32	
2	1	0		90	130		45	30	
3	1	0		90	130		43	30	
4	2	0		85	130		52	34	
5	0.5	0		90	125		40	28	
6	1	0		90	130		44	28	
7	2	0		85	125		50	30	
8	2	0		85	125		52	33	
9	3	0		80	125		57	30	
10	1.5	0		90	125		48	35	
11	2	0		85	125		51	29	
12	2	0		85	125		53	32	
13	2	0		85	125		51	30	
14	3.5	0.5		75	125		63	30	
15	1.5	0		90	130		47	34	
16	2	0		85	125		54	36	
Average (SD)	1.81 (0.75)	0.03 (0.13)		85.6 (4.43)	126.9 (2.50)		50.4 (5.90)	31.3 (2.47)	

LLD, lower limbs discrepancy; NSA, neck-shaft angle. Bold values represent *p* < 0.001.

## Data Availability

The data used to support the findings of this study are available from the corresponding author upon request.
